# Supplementary material for: Pharmacokinetic/Pharmacodynamic (PK/PD) Simulation for Dosage Optimization of Colistin Against Carbapenem-Resistant Klebsiella pneumoniae and Carbapenem-Resistant Escherichia coli
Source: Antibiotics (Basel). 2019 Aug 22;8(3):125. doi: 10.3390/antibiotics8030125 (PMC6784242; doi:10.3390/antibiotics8030125)
Supplement: Supplementary file 1 [file antibiotics-08-00125-s001.pdf]

Supplementary Material

The ranges of creatinine clearance (CrCl) used were ≥80, 50 to 79, 30 to <50, 10 to 29 and <10 mL/min.

Table S1. Colistin dosage regimens for Simulations.

Creatinine Clearance	Daily Dose (CBA)
**EMA-approved regimen[a]**	
≥80 mL/min	150 mg every 12 h
50 to 79 mL/min	150 mg every 12 h
30 to <50 mL/min	125 mg every 12 h
10 to 29 mL/min	150 mg every 24 h
<10 mL/min	120 mg every 24 h
**FDA-approved regimen[b]**	
≥80 mL/min	150 mg every 12 h
50 to 79 mL/min	114 mg every 12 h
30 to <50 mL/min	150 mg every 24 h
10 to 29 mL/min	60 mg every 24 h
<10 mL/min	60 mg every 24 h
**Nation et al. regimen[c]**	
≥80 mL/min	180 mg every 12 h
50 to 79 mL/min	150 mg every 12 h
30 to <50 mL/min	110 mg every 12 h
10 to 29 mL/min	90 mg every 12 h
<10 mL/min	75 mg every 12 h
**Siriraj Hospital regimen**	
>80 mL/min	150 mg every 8-12 h
>40 to 80 mL/min	150 mg every 12 h
>20 to 40 mL/min	100 mg every 12 h
≤20 mL/min	150 mg every 24 h
**Modified from Siriraj Hospital regimen[d]**	
≥80 mL/min	150 mg every 8 h
50 to 79 mL/min	150 mg every 12 h
30 to <50 mL/min	100 mg every 12 h
10 to 29 mL/min	150 mg every 24 h
<10 mL/min	150 mg every 24 h

CBA = colistin base activity, EMA = European Medicine Agency, FDA = US-Food and Drug Administration. A loading dose of 300 mg is given in all regimens. All of regimens were infused over 30 min. a = The European Medicines Agency (EMA) expressed doses in terms of MIU per day. The EMA doses have been converted to approximately equivalent doses expressed as milligrams of CBA and the highest dose in that range was evaluated. For patient with creatinine clearance 10 to 29 mL/min the lowest dose in that range was evaluated. b = For US Food and Drug Administration (FDA)–approved dose suggestions, a body weight of 60 kg was used and the highest dose in that range was evaluated. For patient with creatinine clearance 10 to 29 mL/min, the FDA-approved product label states 1.5 mg CBA/kg every 36 hours, which has been converted in the table to the corresponding daily rate. For patient with creatinine clearance <10 mL/min, the FDA-approved dose suggestions are not stated. For patient with creatinine clearance <10 mL/min, calculations were conducted using a daily dose of CBA of 1 mg/kg as suggested in the FDA-approved product label for patients having creatinine clearance of 10 to 29 mL/min. c = For Nation, et al. dose suggestions, used daily doses of colistin for a desired target colistin Css, avg of 2 mg/L for narrow windows of creatinine clearance and the highest dose (approximately dose) in that range was evaluated. Daily dose administered in 2 divided doses 12 h apart. d = For Siriraj Hospital dose suggestions, Modified from Siriraj Hospital regimen used in simulation.

Table S2. Our study regimens for Simulations.

Creatinine Clearance	Daily Dose (CBA)
**≥80 mL/min**	180 mg every 8 h
**50 to 79 mL/min**	180 mg every 8 h<br>150 mg every 8 h<br>180 mg every 12 h<br>100 mg every 8 h
**30 to <50 mL/min**	180 mg every 8 h<br>150 mg every 8 h<br>180 mg every 12 h<br>150 mg every 12 h<br>100 mg every 8 h
**10 to 29 mL/min**	180 mg every 8 h<br>180 mg every 12 h<br>150 mg every 12 h<br>150 mg every 8 h
**<10 mL/min**	180 mg every 8 h<br>150 mg every 8 h<br>100 mg every 8 h<br>180 mg every 12 h<br>150 mg every 12 h<br>100 mg every 12 h<br>180 mg every 24 h

CBA = colistin base activity, EMA = European Medicine Agency, FDA = US-Food and Drug Administration. A loading dose of 300 mg is given in all regimens. All of regimens were infused over 30 min.

Table S3. Minimum inhibitory concentrations (MICs) distribution of colistin.

MIC (mcg/mL)	0.25	0.5	1	2	4	8	16	32	64	>128	MIC_{50} (mcg/mL)	MIC_{90} (mcg/mL)
**All isolates (n = 116)**	3	55	19	14	4	5	10	4	1	1	0.5	16
%	2.58	47.41	16.38	12.07	3.45	4.31	8.6	3.45	0.86	0.86		
**Colistin-Susceptible Isolates (MIC≤2 mcg/mL)**												
***K. pneumoniae* (n = 74)**	3	40	18	13	-	-	-	-	-	-	0.5	2
***E.coli* (n = 17)**	-	15	1	1	-	-	-	-	-	-	0.5	0.5
**Colistin-Resistant Isolates (MIC > 2 mcg/mL)**												
***K. pneumoniae* (n =22)**	-	-	-	-	3	4	10	3	1	1	16	32
***E. coli* (n =3)**	-	-	-	-	1	1	-	1	-	-	8	32

Table S4. The recommended dose based on the ability to achieve PTA target at various MICs.

Creatinine Clearance (mL/min)	MIC 0.5 mcg/mL Daily Dose (CBA)	MIC 2 mcg/mL Daily Dose (CBA)	MIC 8 mcg/mL Daily Dose (CBA)
**≥80**	150 mg every 12 h (EMA, FDA)	Not recommended	Not recommended
**50-79**	114 mg every 12 h (FDA)	180 mg every 8 h (our study)	Not recommended
**30-<50**	150 mg every 24 h (FDA)	150 mg every 12 h (our study)	Not recommended
**10-29**	60 mg every 24 h (FDA)	150 mg every 12 h (our study)	150 mg every 8 h (our study)

**<10**	60 mg every 24 h (FDA)	120 mg every 24 h (EMA)	180 mg every 12 h (our study)

Table S5. The recommended dose based on the ability to achieve PTA target at various MICs.

**Creatinine Clearance (mL/min)**	**MIC 16 mcg/mL Daily Dose (CBA)**	**MIC 32 mcg/mL Daily Dose (CBA)**
**≥80**	Not recommended	Not recommended
**50-79**	Not recommended	Not recommended
**30-<50**	Not recommended	Not recommended
**10-29**	Not recommended	Not recommended
**<10**	180 mg every 8 h (our study)	Not recommended

MICs = minimum inhibitory concentrations (mcg/mL), EMA = European Medicine Agency, FDA = US-Food and Drug Administration, CBA = colistin base activity.

Table S6. %PTA and %CFR for All isolates, Colistin-susceptible isolates subgroup and Colistin-resistant isolates subgroup.

	**All Isolates**			**Colistin-Susceptible Isolates**							**Colistin-Resistant Isolates**						
			**%CFR**	**Colistin-Susceptible *K. pneumoniae* Subgroup**			**Colistin-Susceptible *E. coli* Subgroup**			**%CFR for all Sus-Ceptible Isolates**	**Colistin-Resistant *K. pneumoniae* Subgroup**			**Colistin-Resistant *E. coli* Subgroup**			**%CFR for all Resistant Isolates**
	**%PTA**			**%PTA**		**%CFR**	**%PTA**		**%CFR**		**%PTA**		**%CFR**	**%PTA**		**%CFR**	
**MIC (mcg/mL)**	**MIC_{50}**	**MIC_{90}**		**MIC_{50}**	**MIC_{90}**		**MIC_{50}**	**MIC_{90}**			**MIC_{50}**	**MIC_{90}**		**MIC_{50}**	**MIC_{90}**		
	**0.5**	**16**		**0.5**	**2**		**0.5**	**0.5**			**16**	**32**		**8**	**32**		
**CrCl ≥80 mL/min**																	
150 mg q12 h (EMA, FDA)	92.66	7.08	70.68	92.66	64.41	85.41	92.66	92.66	90.40	86.34	7.08	1.51	12.45	19.48	1.51	20.33	13.40
180 mg q12 h (Nation et al.)	97.91	8.99	73.14	97.91	69.2	87.87	97.91	97.91	92.25	88.69	8.99	2.2	15.08	24.09	2.2	24.18	16.18
150 mg q8 h (Siriraj)	98.68	19.48	78.56	98.68	79.7	92.02	98.68	98.68	95.06	92.58	19.48	7.02	25.45	39.3	7.02	35.99	26.71
**CrCl 50 to 79 mL/min**																	
114 mg q12 h (FDA)	95.95	9.8	74.90	95.95	71.98	89.91	95.95	95.95	94.07	90.69	9.8	2.36	15.94	25.05	2.36	25.26	17.06
150 mg q12 h (Siriraj, EMA, Nation et al)	97.26	13.73	77.96	97.26	78.32	92.65	97.26	97.26	95.82	93.24	13.73	3.8	20.53	32.72	3.8	31.22	21.81
**CrCl 30 to <50 mL/min**																	
150 mg q24 h (FDA)	97.55	3.58	74.65	97.55	71.1	91..27	97.55	97.55	95.58	92.07	3.58	0.39	10.05	15.59	0.39	18.84	11.10
100 mg q12 h (Siriraj)	98.89	18.09	81.26	98.89	85.65	95.74	98.89	98.89	97.90	96.14	18.09	5.58	25	38.9	5.58	36.58	26.39
110 mg q12 h (Nation et al.)	99.03	20.03	82.17	99.03	87.43	96.35	99.03	99.03	98.19	96.70	20.03	6.41	27.05	42.66	6.41	39.01	28.48
125 mg q12 h (EMA)	99.15	23.65	83.51	99.15	89.8	97.18	99.15	99.15	98.52	97.43	23.65	8.09	30.41	46.87	8.09	42.66	31.87
**CrCl 10 to 29 mL/min**																	
60 mg q24 h (FDA)	96.33	3.57	70.63	96.33	56.2	86.26	96.33	96.33	97.91	87.56	3.57	0.42	8.11	13.11	0.42	14.41	8.87
150 mg q24 h	99.67	11.16	81.45	99.67	89.35	97.44	99.67	99.67	99.61	97.73	11.16	2.02	20.20	33.24	2.02	33.27	21.77

(EMA, Siriraj)																	
90 mg q12 h (Nation et al.)	99.63	11.23	81.45	99.63	89.32	97.41	99.63	99.63	99.93	97.70	11.23	1.94	20.29	33.41	1.94	33.4	21.87
**CrCl <10 mL/min**																	
75 mg q12 h (Nation et al.)	100	50.65	89.32	100	97.96	99.60	100	100	100	99.65	50.65	26.85	53.02	74.32	26.85	63.87	54.32
120 mg q24 h (EMA)	99.34	21.74	84.70	99.94	94.92	98.91	99.94	99.94	99.99	99.04	21.74	5.98	29.99	48.29	5.98	43.72	31.63
150 mg q24 h (Siriraj.)	99.98	28.36	86.51	99.98	97.4	99.47	99.98	99.98	100	99.54	28.36	8.69	36.24	57.76	8.69	50.55	37.96
60 mg q24 h (FDA)	99.5	11.81	78.82	99.5	77.09	94.47	99.5	99.5	99.99	95.11	11.81	2.61	17.88	28.19	2.61	27.44	19.03

MICs = minimum inhibitory concentrations (mcg/mL), PTA = Probability of target attainment, CFR = Cumulative fraction of response, CrCl = Creatinine clearance (ml/min), EMA = European Medicine Agency, FDA = US-Food and Drug Administration.

Table S7. %PTA and %CFR of our study regimens for All isolates, Colistin-susceptible isolates subgroup and Colistin-resistant isolates subgroup.

	All Isolates			Colistin-Susceptible Isolates							Colistin-Resistant Isolates						
			%CFR	**Colistin-Susceptible *K. pneumoniae* Subgroup**			**Colistin-Susceptible *E. coli* Subgroup**			**%CFR for all Sus-Ceptible Isolates**	**Colistin-Resistant *K. pneumoniae* Subgroup**			**Colistin-Resistant *E. coli* Subgroup**			**%CFR for all Resistant Isolates**
	**%PTA**			**%PTA**		**%CFR**	**%PTA**		**%CFR**		**%PTA**		**%CFR**	**%PTA**		**%CFR**	
**MIC (mcg/mL)**	**MIC_{50}**	**MIC_{90}**		**MIC_{50}**	**MIC_{90}**		**MIC_{50}**	**MIC_{90}**			**MIC_{50}**	**MIC_{90}**		**MIC_{50}**	**MIC_{90}**		
	**0.5**	**16**		**0.5**	**2**		**0.5**	**0.5**			**16**	**32**		**8**	**32**		
**CrCl ≥80 mL/min**																	
180 mg q8h	97.19	23.74	80.80	97.19	83.61	93.83	97.19	97.19	96.14	92.58	23.74	9.17	29.55	45.2	9.17	40.54	30.87
**CrCl 50 to 79 mL/min**																	
100 mg q8 h	97.36	23.43	80.60	97.36	82.62	93.77	97.36	97.36	96.23	94.23	23.43	9.4	28.79	43.67	9.4	39.25	30.05
180 mg q12 h	97.82	18.04	80.15	97.82	83.54	94.31	97.82	97.82	96.72	94.76	18.04	5.7	24.88	39.23	5.7	36.31	26.25
150 mg q8 h	98.79	34.52	85.01	98.79	89.88	96.68	98.79	98.79	98.12	96.94	34.52	15.78	38.86	56.93	15.78	49.72	40.16
180 mg q8 h	99.15	39.07	86.51	99.15	92.05	97.44	99.15	99.15	98.61	97.65	39.07	19.36	43.05	61.77	19.36	54.16	44.38
**CrCl 30 to <50 mL/min**																	
100 mg q8 h	99.64	40.8	87.65	99.64	94.49	98.48	99.64	99.64	99.27	98.63	40.8	20.09	44.71	64.06	20.09	55.97	46.06
150 mg q12 h	99.41	27.61	84.91	99.41	92.44	97.84	99.41	99.41	98.91	98.04	27.61	9..97	34.47	53.71	9.97	47.36	36.01
180 mg q12 h	99.67	33.25	86.48	99.67	94.6	98.51	99.67	99.67	99.30	98.66	33.25	13.25	39.30	60.3	13.25	52.09	40.84
150 mg q8 h	99.76	54.13	90.59	99.76	97.29	99.23	99.76	99.76	99.59	99.30	54.13	29.89	55.53	76.01	29.89	65.64	56.75
180 mg q8 h	99.9	60.87	91.96	99.9	97.97	99.44	99.9	99.9	99.76	99.50	60.87	35.86	60.93	81.54	35.86	70.26	62.05
**CrCl 10 to 29 mL/min**																	
150 mg q12 h	99.91	50.76	90.39	99.91	98.35	99.60	99.91	99.91	99.81	99.64	50.76	25.25	53.34	76.03	25.25	64.55	54.68
180 mg q12 h	99.97	57.93	91.71	99.97	99.06	99.78	99.97	99.97	99.91	99.80	57.93	30.34	58.70	81.51	30.34	68.9	59.93
150 mg q8 h	99.96	75.28	94.91	99.96	99.57	99.88	99.96	99.96	99.93	99.89	75.28	52.4	72.87	91.10	52.4	80.38	73.77
180 mg q8 h	100	80.65	95.87	100	99.63	99.92	100	100	99.97	99.93	80.65	59.11	77.09	93.47	59.11	83.57	77.87
**CrCl≤10 mL/min**																	
180 mg q24 h	100	33.88	87.73	100	98.53	99.72	100	100	99.91	99.75	33.88	10.94	40.91	64.74	10.94	54.99	42.60
100 mg q12 h	99.98	59.11	92.02	99.98	99.18	99.83	99.98	99.98	99.93	99.85	59.11	34.22	59.95	81.32	34.22	70.5	61.16
100 mg q8 h	100	81.59	96.14	100	99.79	99.96	100	100	99.99	99.96	81.59	61.67	78.21	93.13	61.67	84.47	78.96

150 mg q12 h	100	71.94	94.13	100	99.84	99.98	100	100	99.99	99.97	71.94	46.32	69.86	89.74	46.32	78.00	70.84
180 mg q12 h	100	77.7	95.30	100	99.90	99.97	100	100	99.99	99.98	77.7	52.76	74.31	92.85	52.76	81.47	75.17
150 mg q8 h	100	89.51	97.76	100	99.97	99.99	100	100	100	99.99	89.51	74.47	85.46	97.26	74.47	90.46	86.06
180 mg q8 h	100	92.31	98.30	100	99.94	99.99	100	100	100	99.99	92.31	78.66	87.97	98.18	78.66	92.19	88.48

MICs = minimum inhibitory concentrations (mcg/mL), PTA = Probability of target attainment, CFR = Cumulative fraction of response, CrCl = Creatinine clearance (mL/min).

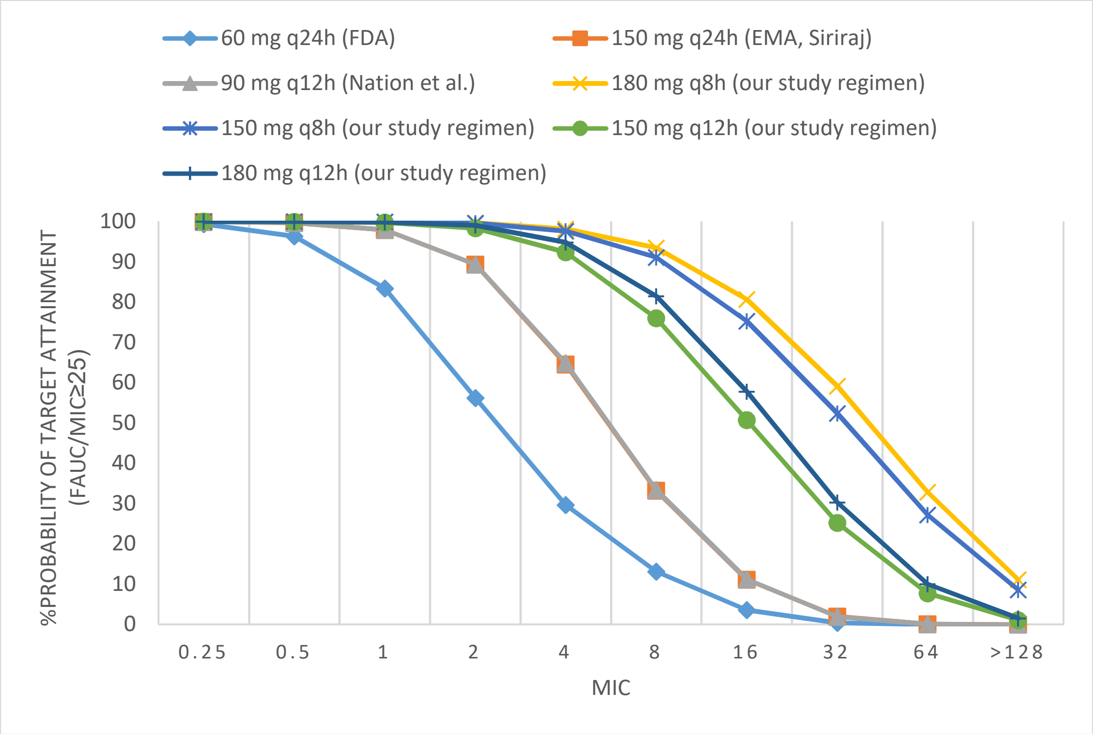

Figure S1. %PTA of colistin in patients with CrCl 10-29 (ml/min).

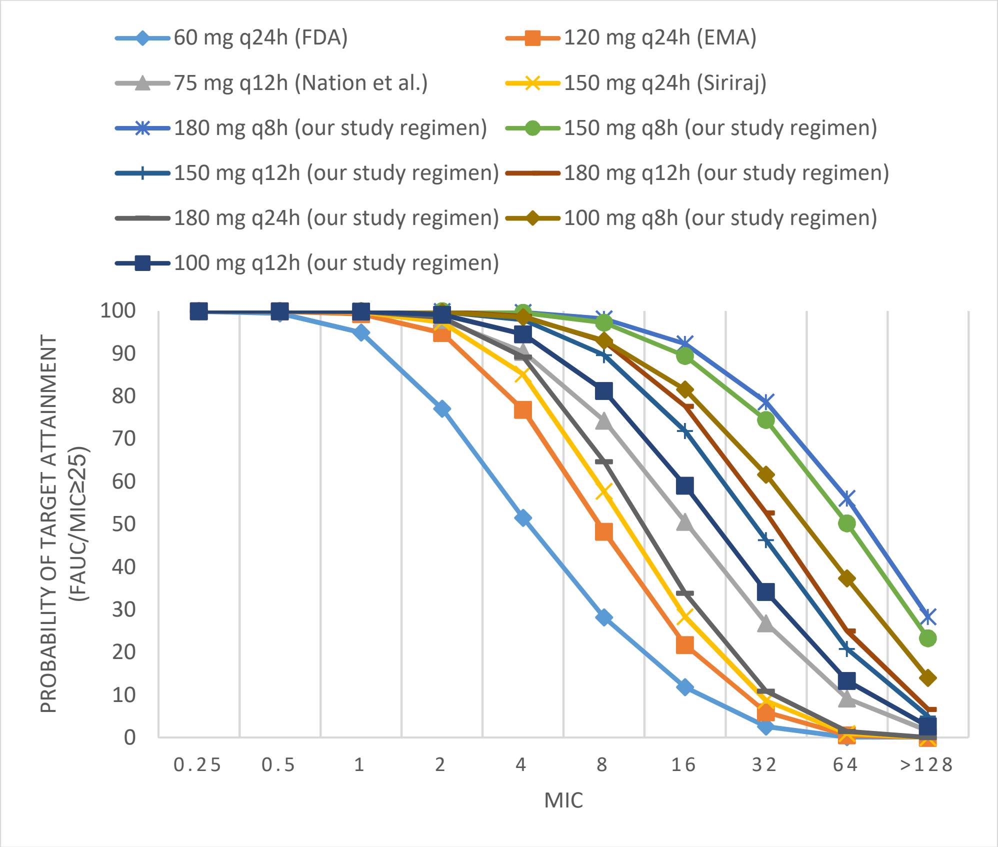

Figure S2. %PTA of colistin in patients with CrCl < 10 (ml/min).
